# Si permeability of a deficient Lsi1 aquaporin in tobacco can be enhanced through a conserved residue substitution

**DOI:** 10.1002/pld3.163

**Published:** 2019-08-21

**Authors:** Devrim Coskun, Rupesh Deshmukh, Humira Sonah, Sheelavanta Matha Shivaraj, Rachelle Frenette‐Cotton, Laurence Tremblay, Paul Isenring, Richard R. Bélanger

**Affiliations:** ^1^ Département de Phytologie, Faculté des Sciences de l’Agriculture et de l’Alimentation (FSAA) Université Laval Québec QC Canada; ^2^ National Agri‐Food Biotechnology Institute (NABI) Mohali Punjab India; ^3^ Nephrology Group, Department of Medicine, Faculty of Medicine, L’Hôtel‐Dieu de Québec Institution Université Laval Québec QC Canada

**Keywords:** aquaporin, influx, Lsi1, molecular determinants, NIP2‐1, silicon (Si), tobacco

## Abstract

Silicon (Si) is a beneficial substrate for many plants, conferring heightened resilience to environmental stress. A plant's ability to absorb Si is primarily dependent on the presence of a Si‐permeable Lsi1 (NIP2‐1) aquaporin in its roots. Structure‐function analyses of Lsi1 channels from higher plants have thus far revealed two key molecular determinants of Si permeability: (a) the amino acid motif GSGR in the aromatic/arginine selectivity filter and (b) 108 amino acids between two highly conserved NPA domains. Curiously, tobacco (*Nicotiana sylvestris*) stands as a rare exception as it possesses an Lsi1 (NsLsi1) with these molecular signatures but is reported as a low Si accumulator. The aim of this study was therefore to identify whether additional determinants influence Si permeability via Lsi1 channels, focusing on the role of residues that differ uniquely in NsLsi1 relative to functional Lsi1 homologs. We observed tobacco indeed absorbed Si poorly (0.1% dw), despite *NsLsi1* being expressed constitutively *in planta*. Si influx measured in NsLsi1‐expressing *Xenopus* oocytes was very low (<13% that of OsLsi1 from rice (*Oryza sativa*) over a 3‐hr time course), which likely explains why tobacco is a low Si accumulator. Interestingly, NsLsi1^P125F^ displayed a significant gain of function (threefold increase in Si influx relative to NsLsi1^WT^), which coincided with a threefold increase in plasma membrane localization *in planta*, as measured by transient expression of GFP constructs in *Nicotiana benthamiana* leaves. These findings thus reveal a novel molecular determinant of Si transport in plants and inform breeding, biotechnological, and agricultural practices to effectively utilize Si in the context of plant resilience to environmental stress.

## 1 INTRODUCTION

Silicon (Si) is not an essential mineral nutrient for higher plants (Epstein, 1994). However, it has been shown to protect plants against a wide range of biotic and abiotic stresses in many species (Coskun et al., 2019a; Fauteux, Chain, Belzile, Menzies, & Bélanger, 2006; Liang, Nikolic, Bélanger, Gong, & Song, 2015; Raven, 1983). Although the biological role of Si and the protective mechanisms *in planta* remain contentious (Coskun et al., 2019a,2019b), Si supplementation has the potential to be an effective strategy to ensure agricultural sustainability in the face of rising food demands and environmental stress (Godfray et al., 2010; Liang et al., 2015; Tilman, Balzer, Hill, & Befort, 2011; Wheeler & von Braun, 2013).

Not all plant species are capable of absorbing Si and benefit from Si supplementation (Deshmukh & Bélanger, 2016; Liang et al., 2015). Based on leaf Si‐content levels, plants have traditionally been categorized as low (<0.5%), moderate (0.5%–1.5%), and high (>1.5% dry weight (dw)) Si accumulators (Handreck & Jones, 1967), which corresponds closely with their phylogenetic distributions (Hodson, White, Mead, & Broadley, 2005). Members of the Poaceae, such as rice (*Oryza sativa*; Ma et al., 2006), wheat (*Triticum aestivum*; Chain, Cote‐Beaulieu, Belzile, Menzies, & Bélanger, 2009), and barley (*Hordeum vulgare*; Chiba, Mitani, Yamaji, & Ma, 2009), are among the highest documented Si accumulators of the higher plants, with Si contents typically ranging between 3% and 5%. By contrast, members of the Brassicaceae, such as Arabidopsis (*Arabidopsis thaliana*; Fauteux et al., 2006; Vivancos, Labbé, Menzies, & Bélanger, 2015) and canola (*Brassica rapa*; Sonah, Deshmukh, Labbe, & Bélanger, 2017), as well as the Solanaceae, such as tomato (*Solanum lycopersicum*; Deshmukh et al., 2015) and potato (*Solanum tuberosum*; Vulavala et al., 2016), are known to be low accumulators. As a result of these apparent conserved properties, attempts have been made to categorize the Si permeability of various species based on genotype (Coskun et al., 2019a; Deshmukh & Bélanger, 2016).

The plasma membrane channel Lsi1 (“low silicon 1,” also denoted as NIP2‐1) is a member of the NIP‐III (nodulin26‐like intrinsic protein‐III) subfamily of aquaporins (AQPs) and is responsible for the primary root uptake of Si from the external environment (Ma et al., 2006; Ma & Yamaji, 2015). In coordination with the Si‐efflux transporter Lsi2 (Ma et al., 2007), Lsi1 mediates Si transport into the xylem, eventually resulting in silica deposition in the aerial parts of the plant (Ma & Yamaji, 2015). Thus far, two key molecular signatures have been found to dictate Si permeability via Lsi1: (a) the amino acid motif GSGR (glycine‐serine‐glycine‐arginine) in the so‐called aromatic/arginine (ar/R) selectivity filter, located in the narrowest region of the extracellular side of the pore (Hove & Bhave, 2011; Mitani‐Ueno, Yamaji, Zhao, & Ma, 2011; Wallace & Roberts, 2004) and (b) precisely 108 amino acids between two highly conserved NPA (asparagine‐proline‐alanine) domains which form the second constriction to the pore (Deshmukh et al., 2015). Hitherto, all plant species found to lack NIP‐III AQPs with these features have been described as low Si accumulators (Coskun et al., 2019a).

Species belonging to the Solanaceae are considered low accumulators owing to an extra amino acid (*i.e.,* 109) between the NPA domains of their Lsi1, rendering these channels Si‐impermeable (Deshmukh et al., 2015). However, a unique scenario emerged upon analyzing the amino acid sequence of tobacco (*Nicotiana sylvestris*; Sierro et al., 2013, 2014). NsLsi1 (NsNIP2‐1) was found to possess the known molecular determinants of Si permeability despite tobacco being reported as a low Si accumulator (Zellner, Frantz, & Leisner, 2011). Thus, we sought to investigate the following research questions: Is NsLsi1 Si‐permeable? If so, why is tobacco nevertheless unable to accumulate Si to levels comparable to other species with functional Lsi1 homologs? If not, are there other molecular determinants that dictate Si permeability in Lsi1? Here, we report the identification of a residue unique to NsLsi1 that alters its Si permeability and cellular localization and thus likely explains why tobacco is a low Si accumulator.

## 2 MATERIALS AND METHODS

### 2.1 Plant material and cultivation

In order to compare its tissue Si content with that of other species, tobacco was grown alongside barley, rice, tomato, soybean (*Glycine max*), pigeon pea (*Cajanus cajan*), and poplar (*Populus trichocarpa*). In addition, wheat (*Triticum aestivum*) was grown for the purpose of cloning *TaTIP2‐1* (see below). All species were grown from seed except for poplar, which was grown from wood cuttings (Deshmukh et al., 2015). Surface sterilization of all seeds was performed in 1% sodium hypochlorite for 5 min, followed by several washes in distilled water. Plants were grown in 8‐inch pots filled with a pasteurized soil mix of Pro‐mix:sand:garden soil (4:1:1 ratio by volume) for one month in a climate‐controlled greenhouse (25 ± 2°C, photoperiod of 16 hr). Plants were continuously irrigated with fertilizer (NPK 20:20:20) supplemented with 1.7 mM Si in the form of potassium silicate (Deshmukh et al., 2015).

### 2.2 Tissue Si quantification

To determine tissue Si content, the oldest two to three leaves (composing one replicate) from each one‐month‐old plant were harvested and oven‐dried at 65°C for 24 hr. A total of five biological replicates were collected per species. Dried leaf samples were ground to a fine powder using a mixer mill. From there, the leaf powder was compressed into pellets (13 mm diameter × 5 mm thickness) and subjected to Si quantification using a portable X‐ray fluorescence (XRF) analyzer (Niton XL3t GOLDD XRF Analyzer, Thermo Fisher Scientific). A standard curve for Si estimation was prepared, and Si quantification of samples was performed as previously described (Deshmukh et al., 2015; Reidinger, Ramsey, & Hartley, 2012).

### 2.3 Phylogeny of Lsi1 homologs

Protein sequences of several plant Lsi1 (NIP2‐1) homologs (Deshmukh, Sonah, & Bélanger, 2016; Deshmukh et al., 2015; Montpetit et al., 2012) were subjected to multiple sequence alignments using CLUSTALW implemented in MEGA7 (Kumar, Stecher, & Tamura, 2016). A phylogenetic tree was also developed with the maximum‐likelihood method (1,000 bootstraps).

### 2.4 *NsLsi1* expression analysis

Tobacco plants were grown, as above, with and without Si for 1 month. Total RNA was isolated thereafter from root and shoot tissues in four biological and two technical replications using the Qiagen RNeasy Mini Kit (Qiagen, Hilden, Germany). cDNA was synthesized by oligo(dT) priming from 3 μg of this preparation with the Superscript‐II kit (Thermo Fisher Scientific) and subjected to quantitative PCR (qPCR) using a Mic qPCR Cycler system (Bio Molecular Systems; Queensland, Australia) and the iQ SYBR Green Supermix (Bio‐Rad Laboratories). The expression of *NsLsi1* was analyzed using Mic's RQ software by REST method (Bio Molecular Systems), and *NsActin* and *NsEF1A1* (elongation factor 1‐alpha 1) were used as controls for expression normalization (primers provided in Table S1).

### 2.5 Gene cloning

Total RNA from one‐month‐old root tissues was isolated as described above for tobacco, rice, and wheat. cDNAs were synthesized by oligo(dT) priming from 3 µg of RNA using Superscript III reverse transcriptase (Invitrogen). Genes of interest (*NsLsi1*, *OsLsi1*, and *TaTIP2‐1*) were PCR‐amplified subsequently using cDNA templates, gene‐specific primers (Table S1), and the Phusion Taq polymerase (New England Biolabs) (for details, see Deshmukh et al., 2015).

### 2.6 Heterologous expression in *Xenopus laevis* oocytes and Si influx

The amplified ORF of *NsLsi1*, *OsLsi1*, and *TaTIP2‐1* was cloned in the Pol1 vector for heterologous expression in *Xenopus laevis* oocytes as previously described (Carpentier et al., 2016). After linearization with SphI, the constructs were used as templates to synthesize complementary RNA (cRNA) using the mMESSAGE mMACHINE T7 ULTRA kit (Thermo Fisher Scientific).

Oocytes (all at stage 5 or 6) were harvested from adult *X. laevis,* manually defolliculated, and injected with 25 nl of 2 µg µl^−1^ cRNA or equal volume of MilliQ H_2_O as negative control (Carpentier et al., 2016). Oocytes were then incubated for 24 hr at 18°C in modified Barth's solution (MBS: 88 mM NaCl, 15 mM HEPES, 2.4 mM NaHCO_3_, 1 mM KCl, 0.82 mM MgSO_4_, 0.41 mM CaCl_2_, 0.33 mM Ca(NO_3_)_2_∙4H_2_O, pH 7.4).

For Si‐influx assays, oocytes were incubated (10 oocytes per replicate) in MBS supplemented with 2 mM Si (Carpentier et al., 2016) for varying times at room temperature, after which they were washed three times in a 4°C solution of 0.32 M sucrose and 5 mM HEPES (pH 7.4). To study the dependence of Si transport on external Si, Si‐influx assays were performed as above but in the presence of varying concentrations of Si (using *N*‐methyl‐*D*‐glucamine gluconate as a substitute of Si). Osmolality of all uptake and wash solutions (*c*. 200 mOsm) were measured by an osmometer (model 3,320, Advanced Instruments).

After washing, concentrated nitric acid (25 µl) was added to each replicate of 10 oocytes and dried for 5 hr at 82°C. Plasma‐grade water (100 µl) was subsequently added to the resulting pellets, vortexed, and centrifuged for 10 min at 14,500 ***g***. Intracellular Si concentration was measured in 10 µl of supernatant by a Zeeman atomic absorption spectrometer AA240Z (Varian) equipped with a GTA120 Zeeman graphite tube atomizer (Garneau, Marcoux, Frenette‐Cotton, Bélanger, & Isenring, 2018). The standard curve was obtained using a 1,000‐ppm ammonium hexafluorosilicate solution (Thermo Fisher Scientific). Data were analyzed with the SpectrA software (Varian) and GraphPad Prism 8 software (GraphPad Software).

For data presentation, Si‐content values of H_2_O‐injected oocytes (negative controls) were subtracted from those of Lsi1‐expressing oocytes. When Si transport was analyzed as a function of incubation time or external Si concentration, data were normalized to the highest value at the longest time point or highest concentration. Data are expressed as means ± SE of normalized values from a minimum of nine replicates over three independent experiments. Uptake kinetics were fitted to a Michaelis–Menten regression, and kinetic parameters (*V*_max_ and *K*_m_, ±SE) were calculated using GraphPad Prism 8.

### 2.7 Site‐directed mutagenesis

An extensive amino acid sequence alignment of previously characterized Lsi1 homologs (Deshmukh et al., 2015) revealed that a few residues within the inter‐NPA domain could account for the inability of NsLsi1 to act as a Si‐permeable channel (see Section 3). The SDPpred bioinformatics tool (bioinf.fbb.msu.ru/SDPpred/) was used to select amino acid variations that may determine functional differences between unique Lsi1 groups (Deshmukh et al., 2015; Kalinina, Novichkov, Mironov, Gelfand, & Rakhmaninova, 2004), and the PROVEAN (Protein Variation Effect Analyzer) tool was used to predict the impact of amino acid changes on protein function (http://provean.jcvi.org/index.php; Choi, Sims, Murphy, Miller, & Chan, 2012). Based on this analysis, the variant residue in position 125, a proline (P) in NsLsi1 and phenylalanine (F) in other species, was targeted for further studies (for details, see Section 3).

A site‐directed mutagenesis approach was undertaken to perform the P125F substitution in NsLsi1 (for primer, see Table S1) using the Phusion Taq polymerase (New England Biolabs). The mutated construct was selected for through DpnI restriction and transformed in *E. coli* TOP10 competent cells (see Deshmukh et al., 2015, for details). The mutation was confirmed by automated sequencing.

### 2.8 Leaf transient expression analysis

To obtain GFP‐tagged NsLsi1^WT^ and NsLsi1^P125F^ constructs for heterologous expression *in planta*, the coding region (minus stop codon) of NsLsi1^WT^ and NsLsi1^P125F^ was amplified using gene‐specific primers containing BamHI and SpeI restriction sites (Table S1), and amplicons were cloned through these sites into the pCAMBIA1302 vector (CAMBIA; Canberra, Australia), which included (from 5′ to 3′) the 35S promoter, the gene of interest, and the in‐frame fused GFP‐coding sequence. The pCAMBIA1302 vector carrying only the GFP gene under the 35S promoter (35S::GFP construct) served as a negative control. Constructs were verified by automated sequencing and used to transform *Agrobacterium tumefaciens* GV3101 (pMP90) competent cells.

Transient expression in *Nicotiana benthamiana* leaves was performed as previously described (Johansen and Carrington, 2001) by mixing transformants with *A. tumefaciens* pGD‐p19 cultures on the abaxial surface of leaves. The pGD‐p19 plasmid expresses the Tomato Bushy Stunt Virus (TBSV) p19 coding region to reduce host RNA silencing (Bragg & Jackson, 2004). Leaves were harvested five days post‐agroinfiltration and assayed for cellular localization through confocal laser scanning microscopy (40x objective; Model FV1000, Olympus). In all cases, individual cells were chosen at random. Fluorescence intensity was measured in ImageJ (FIJI Plugin) by drawing two bisecting lines across the centre of the cell and measuring the two peak fluorescent intensities (in arbitrary units) per line (corresponding to the plasma membrane; Figure S1). Imaging experiments were performed and analyzed in a blinded manner. Data are expressed as means ± SE of six cells over three plants (NsLsi1^WT^) and 17 cells over four plants (NsLsi1^P125F^) and analyzed by Student's *t* test.

## 3 RESULTS

### 3.1 Tissue Si quantification and Lsi1 phylogeny

**Figure 1:**
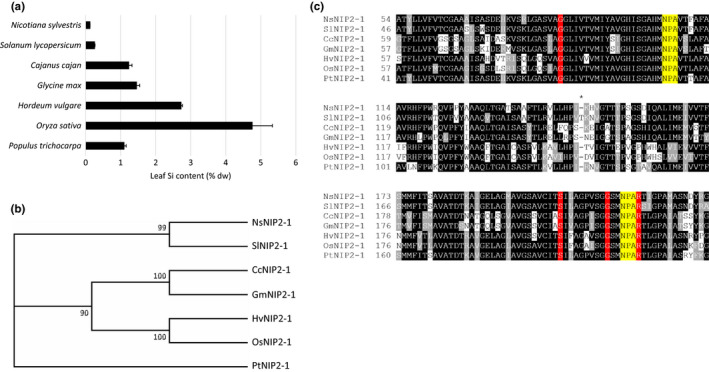
Tobacco is a low Si accumulator despite possessing the known molecular determinants for Si permeability. (a) Leaf Si content (% dw) of one‐month‐old plants grown with 1.7 mM Si supplementation. Error bars denote *SEM* of five biological replicates. (b) Phylogenetic tree of Lsi1 (NIP2‐1) AQPs corresponding to the species from panel a. (c) Amino acid sequence alignment of Lsi1 AQPs corresponding to panel b. Residues highlighted in red denote the positions of the ar/R selectivity filter. Residues highlighted in yellow denote the positions of the NPA domains. 108 amino acids separate the NPA domains of all sequences except for SlNIP2‐1, which has an extra amino acid located at the position of the asterisk

Figure 1a shows the leaf Si content (% dw) of tobacco in comparison with a range of species known to differ in their Si‐accumulating properties. Tobacco accumulated the lowest Si content (0.1%), followed by tomato (0.2%). By contrast, pigeon pea, soybean, barley, and poplar accumulated between 1% and 3% Si, and rice, over 4.5%.

Tissue Si content followed a clear phylogenetic distribution based on the Lsi1 (NIP2‐1) homologs of these species (Figure 1b). NsNIP2‐1 and SlNIP2‐1 from tobacco and tomato, respectively, formed a clade, as did CcNIP2‐1 and GmNIP2‐1 from pigeon pea and soybean, respectively, and OsNIP2‐1 and HvNIP2‐1 from rice and barley, respectively. PtNIP2‐1 from poplar was independent.

Figure 1c shows the results of an amino acid sequence alignment of the Lsi1 proteins belonging to the species above. Here, Lsi1 homologs possessing a GSGR selectivity filter and 108 amino acids between their NPA domains belonged to species which accumulated Si greater than 1%. SlNIP2‐1 possesses 109 amino acids between its NPA domains, and, as a result, has been shown to lack Si permeability (Deshmukh et al., 2015). Curiously, the sole exception to this genotype–phenotype relationship was tobacco (NsLsi1), which possesses a GSGR motif and 108 amino acids between its NPA domains, suggestive of a Si‐permeable Lsi1, despite accumulating very little Si *in planta* (Figure 1a).

As an additional control, and to further confirm that NsLsi1 was an exception, the H_2_O‐ and NH_3_‐permeable TIP (tonoplast intrinsic protein) AQP, TaTIP2‐1 from wheat (possessing an HIGR motif in its ar/R selectivity filter and 111 amino acids spanning its NPA domains; Hove & Bhave, 2011; Jahn et al., 2004), was tested. This channel was also found to lack Si permeability (Fig. S2).

### 3.2 *NsLsi1* expression *in planta*

**Figure 2:**
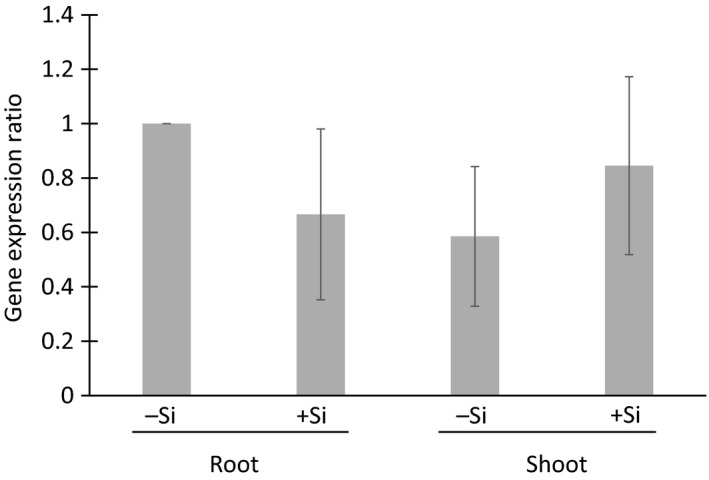
*NsLsi1* expression *in planta*. qPCR results showing *NsLsi1* expression in roots and shoots of tobacco plants grown for one month with (+Si) and without (−Si) silicon. Gene expression was normalized to *NsActin* and *NsEF1A1.* Error bars denote *SEM* of four biological and two technical replicates

To determine whether *NsLsi1* was expressed *in planta*, and whether gene expression was regulated by the presence of Si itself, qPCR was performed on whole roots and shoots of one‐month‐old tobacco plants that were grown with and without Si. Figure 2 shows that, indeed, *NsLsi1* was expressed constitutively throughout the plant, independent of plant Si status.

### 3.3 Heterologous expression in *Xenopus laevis* oocytes and Si influx

**Figure 3:**
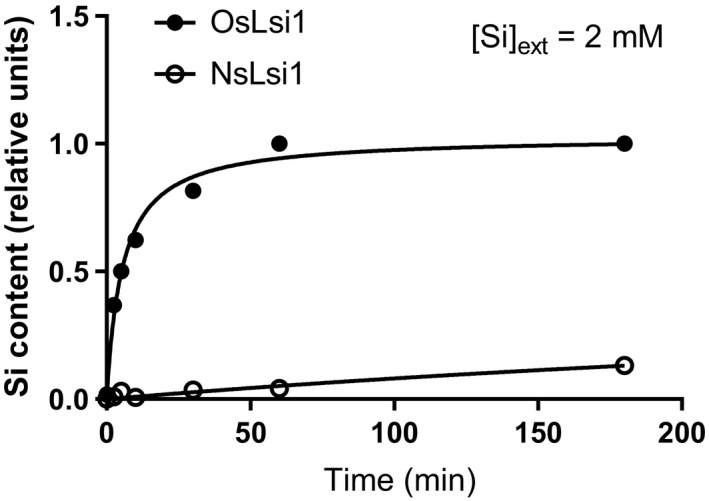
NsLsi1 possesses limited Si‐uptake capabilities. Si content of *Xenopus laevis* oocytes expressing OsLsi1 (filled symbols) or NsLsi1 (open symbols) as a function of exposure time to Si in the external medium ([Si]_ext_ = 2 mM). Si‐content values were corrected for background Si and normalized to the highest time value (i.e., OsLsi1 at 180 min; see text for details). Data represent means of nine replicates (error bars were smaller than symbols) and fitted to a Michaelis–Menten regression

To determine the Si‐transport capabilities of NsLsi1, a time course of Si uptake in *Xenopus* oocytes expressing NsLsi1 was conducted relative to oocytes expressing the positive control OsLsi1. OsLsi1 provided oocytes with rapid and sizeable Si uptake, reaching half‐maximal Si content by 5 min (Figure 3). By contrast, NsLsi1 provided oocytes with no significant Si uptake by 5 min (*p* > .05, relative to H_2_O‐injected controls), and only minimal uptake by 3 hr (13% the Si content of OsLsi1‐expressing oocytes).

### 3.4 Site‐directed mutagenesis

**Figure 4:**
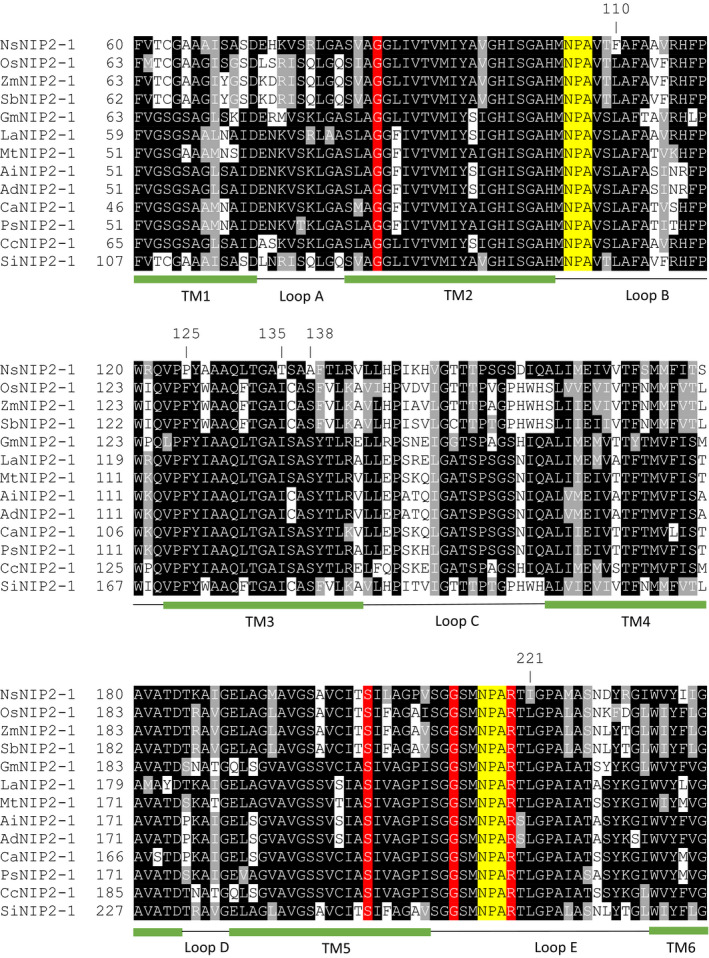
Amino acid sequence alignment of Lsi1 homologs. Residues marked by a position (based on the sequence of NsNIP2‐1) are unique to this homolog and indicate potential candidates for mutagenesis (see text and Table 1 for details). Residues composing the ar/R selectivity filter are highlighted in red and those composing the NPA domains in yellow. The number of amino acids spanning the NPA domains for all proteins is 108. Green bars denote the position of the six transmembrane (TM) helices, interspersed by five interconnecting loops (A–E) denoted by black lines. NsNIP2‐1 from tobacco (*Nicotiana sylvestris*), OsNIP2‐1 from rice (*Oryza sativa*), ZmNIP2‐1 from maize (*Zea mays*), SbNIP2‐1 from sorghum (*Sorghum bicolor*), GmNIP2‐1 from soybean (*Glycine max*), LaNIP2‐1 from blue lupin (*Lupinus angustifolius*), MtNIP2‐1 from barrelclover (*Medicago truncatula*), AiNIP2‐1 and AdNIP2‐1 from peanut ancestors *Arachis ipaensis* and *Arachis duranensis*, respectively, CaNIP2‐1 from chickpea (*Cicer arietinum*), PsNIP2‐1 from pea (*Pisum sativum*), CcNIP2‐1 from pigeon pea (*Cajanus cajan*), and SiNIP2‐1 form foxtail millet (*Setaria italica*)

An extensive amino acid sequence alignment comparing NsLsi1 with several functional Lsi1 homologs was conducted to determine whether NsLsi1 lacked any conserved residues which could potentially contribute to its lack of Si‐transport capability (Figure 4). Five such residues were found to be unique to NsLsi1 while being highly conserved among all other proteins, including OsLsi1: F110L, P125F, T135I, A138S, and I221L.

Using SDPpred, each potential substitution variant for NsLsi1 (and in reverse for OsLsi1) was further analyzed for its predicted effect on protein function (Table 1). Of all the substitution variants, only F128P in OsLsi1 was considered “deleterious” (i.e., with a PROVEAN score below the cutoff value of −2.5; Choi et al., 2012), with all others considered “neutral” (i.e., with a PROVEAN score above −2.5). Given the unique case of F128P in OsLsi1, it was determined that the corresponding P125F substitution in NsLsi1 should be considered for its functional effects.

**Table 1:** Predicted effect of amino acid substitutions between NsLsi1 and OsLsi1 proteins. A Protein Variation Effect Analyzer (PROVEAN) score less than or equal to −2.5 was considered “deleterious” and above −2.5 was considered “neutral” (see text for details)

NsLsi1 variant	PROVEAN score	Prediction	OsLsi1 variant	PROVEAN score	Prediction
F110L	2.498	Neutral	L113F	−2.393	Neutral
P125F	1.994	Neutral	F128P	−2.650	Deleterious
T135I	0.247	Neutral	I138T	−1.736	Neutral
A138S	0.960	Neutral	S141A	−0.844	Neutral
I221L	0.940	Neutral	L224I	−1.026	Neutral

**Figure 5:**
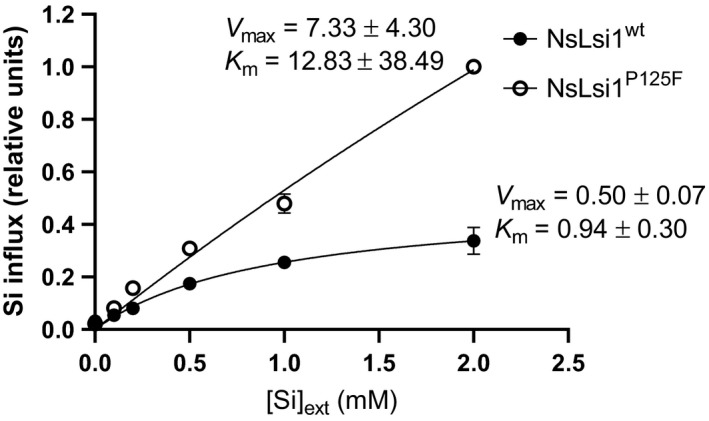
Kinetic analysis of Si influx mediated by NsLsi1^WT^ and NsLsi1^P125F^. Si influx in *Xenopus laevis* oocytes expressing NsLsi1^WT^ (filled symbol) or NsLsi1^P125F^ (open symbol) as a function of external Si concentration ([Si]_ext_). Exposure time = 3 hr. Si influx was corrected for background Si and normalized to the highest concentration value (i.e., NsLsi1^P125F^ at 2 mM Si; see text for details). Error bars denote *SEM* of nine replicates. Data were fitted to a Michaelis–Menten regression

Figure 5 shows the Si‐influx kinetics of NsLsi1^WT^‐ and NsLsi1^P125F^‐expressing oocytes as a function of external Si concentration ([Si]_ext_). The P125F substitution conferred a significant gain of function to NsLsi1 that was observed at [Si]_ext_ as low as 0.2 mM (*p* < .01) and that amounted to a threefold increase at 2 mM. The [Si]_ext_‐dependence of Si influx was fitted by a Michaelis–Menten regression for both proteins (WT: *V*_max_ = 0.50 ± 0.07, *K*_m_ = 0.94 ± 0.30, *R* ^2^ = 0.90; P125F: *V*_max_ = 7.33 ± 4.30, *K*_m_ = 12.83 ± 8.49, *R* ^2^ = 0.98), although it was best fitted by a linear equation for the mutant (*R* ^2^ = 0.99).

To test the hypothesis that the gain of function observed in the NsLsi1^P125F^‐expressing oocytes was as a result of elevated plasma membrane expression of the protein, we added a cMyc tag to both constructs to carry out Western blot analyses (data not shown). Unfortunately, the tagged proteins were found to be nonfunctional (i.e., Si‐impermeable) and thus could not be exploited further. Attempts at overcoming these issues by using other epitopes (HA and FLAG) at either extremity (C vs. N terminus) did not resolve this issue. The methods used to epitope‐tag NsLsi1 variants are outlined in Supplemental Materials.

### 3.5 Leaf transient expression analysis

**Figure 6:**
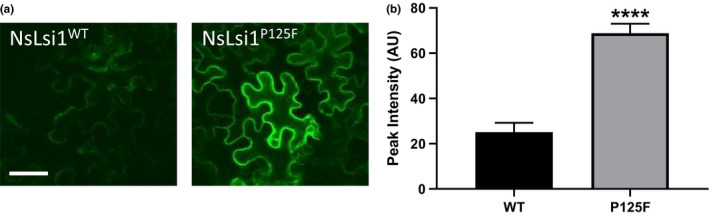
NsLsi1^P125F^ displays enhanced plasma membrane localization relative to wild‐type. (a) Confocal micrographs displaying cellular localization of GFP fused to the C terminus of NsLsi1 wild‐type (left) or P125F mutant (right) in an *Nicotiana benthamiana* leaf transient expression assay. Scale bar denotes 50 μm. (b) Quantitative analysis of fluorescence intensity at the plasma membrane (*n* = 6–17; ****p < .0001, Student's *t* test)

Considering the issues above, we proceeded to investigate the transient expression of GFP‐tagged NsLsi1^WT^ and NsLsi1^P125F^ in *Agrobacteria*‐infiltrated *N. benthamiana* leaves. Figure 6a shows that compared to NsLsi1^WT^, NsLsi1^P125F^ is indeed expressed at much higher levels at the plasma membrane. Fluorescence intensity at the plasma membrane was found to be threefold higher in NsLsi1^P125F^ relative to NsLsi1^WT^ (*p* < .0001; Figure 6b).

## 4 DISCUSSION

Tobacco is known to be a poor Si accumulator, as observed for other Solanaceae (Deshmukh et al., 2015), but has recently drawn attention as a puzzling case because of its residue composition. We had previously observed that Lsi1 (NIP2‐1) channels with a GSGR motif in the ar/R selectivity filter and precisely 108 amino acids between the NPA domains were Si‐permeable and therefore conferred plants the ability to accumulate Si at levels exceeding 1% (Coskun et al., 2019a; Deshmukh & Bélanger, 2016; Deshmukh et al., 2015). However, as we report here, NsLsi1 possesses these molecular characteristics and yet tobacco remains a poor Si accumulator (<0.1% dw).

Here, we showed that in the *Xenopus* oocyte heterologous expression system, NsLsi1^WT^ does not transport Si to an appreciable extent, particularly in comparison with OsLsi1. This observation alone leads to several interesting interpretations. First, it offers a strong inference as to why tobacco is unable to accumulate Si *in planta*; that is, it corroborates the notion that a functional Lsi1 is a key factor in determining the ability of a plant to absorb Si from the external environment. Second, it validates the efficacy of the *Xenopus* oocyte system at assessing the permeability of AQPs to various substrates. This is all the more relevant as our study not only showed the selective permeability of NIP‐III AQPs for Si, but further showed that water‐permeable AQPs such as TaTIP2‐1 did not allow the passage of Si, as we similarly observed in the case of human AQPs (Garneau et al., 2015), and offers additional evidence that the entry of Si into plants is not determined by water fluxes (Coskun et al., 2019a). Lastly, it suggests that molecular determinants other than the ar/R selectivity filter and inter‐NPA distance can affect the Si permeability of Lsi1 channels.

Following comparative amino acid alignments of NsLsi1 with several functional Lsi1 homologs, we found five residues in NsLsi1 that were at odds with highly conserved residues at their respective positions, and thus potential causal variants of the functional differences observed in vivo. Of these, only one, residue P125, was predicted to alter protein function (based on the deleterious effect, this residue was predicted to have upon substitution in OsLsi1) and was thus subsequently retained for further analyses. Interestingly, its potential to alter protein function was confirmed by showing that substitution of this amino acid for the conserved one, that is, P125F, translated into a significant gain of function in Si transport.

Curiously, Western blot analyses were unable to address the hypothesis that the gain of function was as a result of increased plasma membrane localization of the mutant protein, as in vivo function was lost in both WT and P125F constructs. It is possible that the epitopes produced a conformational effect on NsLsi1 that prevented antibody recognition. Alternatively, the epitopes may have interfered with the normal cellular trafficking of the NsLsi1 variants, leading to degradation or endoplasmic reticulum retention (Jarvik & Telmer, 1998). Importantly, expression of the GFP‐tagged mutant protein *in planta* was clearly higher relative to the GFP‐tagged WT protein. Thus, this suggests, albeit indirectly, that the gain of function observed in oocytes is possibly the result of increased protein abundance at the plasma membrane.

An increase in the number of transporters at the plasma membrane could certainly contribute to the altered transport kinetics we observed in NsLsi1^P125F^ relative to NsLsi1^WT^, particularly with respect to increased flux capacity (*V*_max_). Although merely correlative, it is interesting to note that the P125F substitution resulted in both a threefold increase in Si influx (at 2 mM [Si]_ext_) and plasma membrane expression (as measured by fluorescence intensity). Moreover, the data indicate potential structural changes also occurred that could influence substrate affinity (K_m_). Whereas the WT displayed modest and saturable kinetics, the P125F construct displayed a sizeable and linear response to rising [Si]_ext_. It is important to note, however, that such isotherms are complicated by the fact that the solubility of silicic acid (Si(OH)_4_), the bioavailable and permeating Si species, saturates at *c*. 2 mM (Ma, Miyake, & Takahashi, 2001). Thus, although Si influx via NsLsi1^P125F^ may be linear here, it will certainly be limited by chemical constraints before any biological constraints.

The P125 residue in NsLsi1 is found near the beginning of the third transmembrane helix (TM3), just after loop B, and is predicted to face the cytosol. It is thus present in a domain that has access to cargo proteins, regulatory enzymes, cytoskeletal elements, and chaperones, all of which are known to play an important role in membrane localization (Reyes, Buono, & Otegui, 2011). Additionally, proline is a unique residue that is found at turning points within the secondary structure of proteins because of its very short pyrrolidine side chain. It could thus prevent loop B and the early TM3 domain from driving membrane expression through relevant interactions. Interestingly, proline residues have been found to play important roles (both positive and negative) in the expression of transporters (Paczkowski & Bryan‐Lluka, 2004; Slepkov, Chow, Lemieux, & Fliegel, 2004). Furthermore, it is noteworthy that SlLsi1 from tomato also lacks the highly conserved phenylalanine residue in the position of interest, but instead of a proline (as in NsLsi1), it contains a valine. As previously observed (Deshmukh et al., 2015), the deficient Si transport of SlLsi1 could be significantly elevated by a single deletion reducing the number of inter‐NPA residues from 109 to 108, the latter corresponding to all Si‐permeable Lsi1 channels to date. This suggests that the valine of SlLsi1, corresponding to the P125 residue in NsLsi1, does not affect Si permeability, at least not as strongly as proline, although the evolutionary basis for this unique feature within Solanaceae warrants further research.

A similar scenario was found in the case of CmLsi1 from pumpkin (*Cucurbita moschata*), wherein two rootstock cultivars differing in Si‐uptake capabilities corresponded with contrasting Si content in cucumber grafts (Mitani, Yamaji, Ago, Iwasaki, & Ma, 2011). A single amino acid, a proline at position 242, segregated with the functional (i.e., Si‐permeable) CmLsi1 variant, whereas a leucine in this position segregated with a loss‐of‐function phenotype. The latter was also explained by an inability of the transporter to localize to the plasma membrane. As far as we can tell, the L242 residue is only observed in this nonfunctional isoform of CmLsi1, whereas all other Lsi1 homologs (including NsLsi1) possess the P242 residue. Similarly, the P125 residue of NsLsi1 appears unique to tobacco.

These findings raise interesting questions about the evolutionary history of the Solanaceae, in terms of their Si‐accumulating properties, or lack thereof. Whereas the low Si content in species such as tomato could be explained by a Si‐impermeable yet plasma‐membrane‐bound Lsi1, owing to an extra amino acid in the inter‐NPA region (which is also the case in other Solanaceae species, including potato and currant tomato (*Solanum pimpinellifolium*); Deshmukh et al., 2015), it appears the low Si content in tobacco is explained by a completely different mechanism, namely reduced Lsi1 plasma membrane expression, at least in part due to the P125 residue. Indeed, other tobacco species such as *N. tabacum* and *N. benthamiana* also possess Lsi1 homologs with similar molecular characteristics (i.e., a GSGR selectivity filter, 108‐amino acid inter‐NPA distance, and the P125 residue), and presumably lack Si permeability for the same reason. The selective pressures resulting in the low Si content of the Solanaceae and the causes of divergent mechanisms to manifest across the different species remain speculative.

Through this work, we have identified a novel determinant of Si transport via Lsi1. Although this determinant appears to be highly conserved among many species, it has nevertheless pointed us toward a domain of Lsi1 that could play a key role in membrane expression and function. This domain could become a molecular target toward improving Lsi1 channel activity through increased protein delivery, thus potentially increasing the Si content of species, and ultimately elevating plant resilience. This is particularly timely and important, given the needs to improve agricultural sustainability in the face of rising food demands and environmental stress.

## CONFLICT OF INTEREST

The authors declare no conflict of interest associated with the work described in this manuscript.

## AUTHORS CONTRIBUTION

DC, RD, SMS, PI, and RRB designed the study. DC, RD, HS, SMS, RF, and LT performed the experiments. All authors contributed to data analysis. DC wrote the manuscript, with input from PI and RRB.
