# Supplementary material for: Si permeability of a deficient Lsi1 aquaporin in tobacco can be enhanced through a conserved residue substitution
Source: Plant Direct. 2019 Aug 21;3(8):e00163. doi: 10.1002/pld3.163 (PMC6702468; doi:10.1002/pld3.163)
Supplement: Supplementary file 1 [file PLD3-3-e00163-s005.pdf]

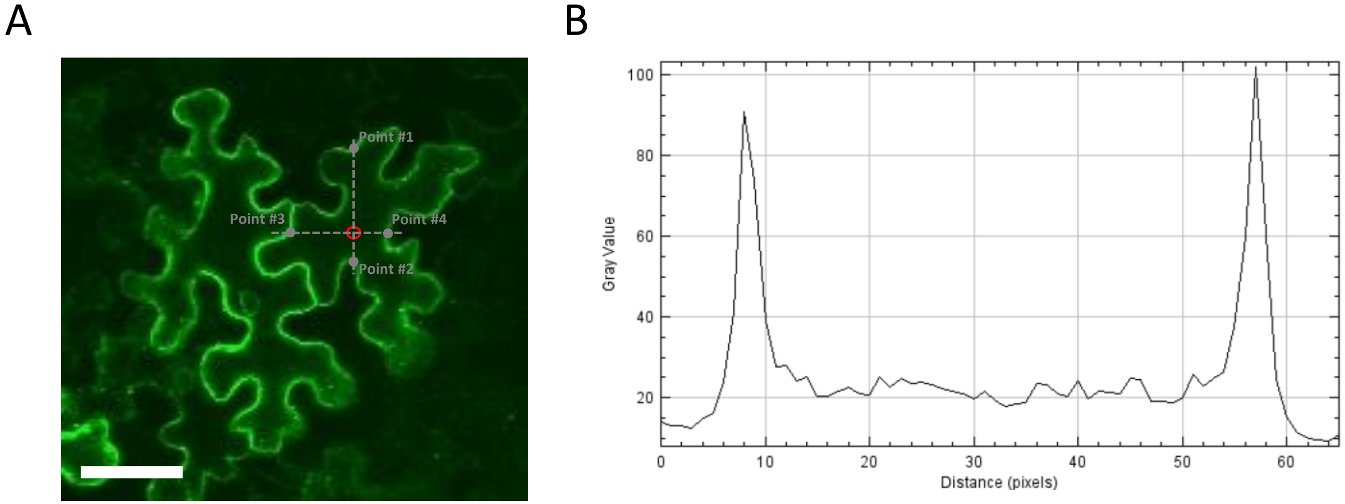

Figure S1. Quantitative analysis of GFP fluorescence. (A) Representative confocal micrograph displaying cellular localization of GFP fused to the C-terminus NsLsi1^{P125F} in an *N. benthamiana* leaf transient expression assay. Scale bar denotes 50 µm. Pixel intensity of perpendicular lines bisecting the centre of the cell was measured with ImageJ software. (B) Representative readout of the pixel intensity of the lines drawn as in panel A.
