# Supplementary material for: Si permeability of a deficient Lsi1 aquaporin in tobacco can be enhanced through a conserved residue substitution
Source: Plant Direct. 2019 Aug 21;3(8):e00163. doi: 10.1002/pld3.163 (PMC6702468; doi:10.1002/pld3.163)
Supplement: Supplementary file 2 [file PLD3-3-e00163-s004.pdf]

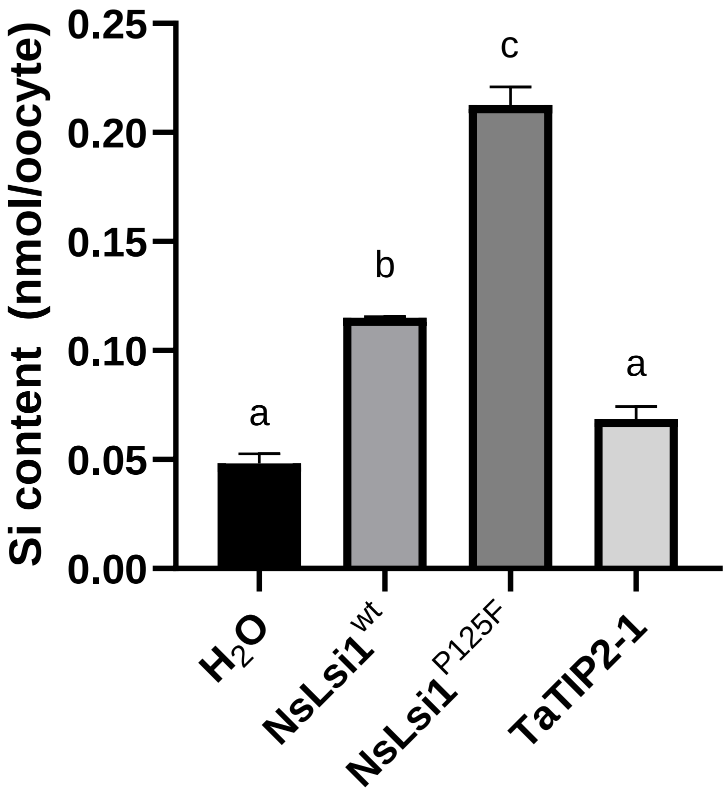

Figure S2. TaTIP2-1 is not a functional Si channel. Si content of *Xenopus laevis* oocytes expressing NsLsi1^{WT}, NsLsi1^{P125F}, and TaTIP2-1 from wheat (*Triticum aestivum*) and exposed to 2 mM Si for 180 min. Water-injected oocytes serve as a negative control. Error bars denote the standard error of three biological replicates. Different letters denote statistically different means ($p < 0.05$, one-way ANOVA with Tukey post-hoc test).
