# Supplementary material for: Si permeability of a deficient Lsi1 aquaporin in tobacco can be enhanced through a conserved residue substitution
Source: Plant Direct. 2019 Aug 21;3(8):e00163. doi: 10.1002/pld3.163 (PMC6702468; doi:10.1002/pld3.163)
Supplement: Supplementary file 3 [file PLD3-3-e00163-s003.pdf]

Supplementary Material

Western Blot Analysis

Western blot analyses were performed as previously described (Carpentier *et al.*, 2016). In brief, oocytes (~80 per assay) were placed in modified Barth's solution (MBS; see Materials and Methods) supplemented with 2 mM sulfo-NHS biotin for 30 min at room temperature to label cell-surface proteins. The reaction was terminated with several washes in MBS supplemented with 10 mM glycine. Subsequently, oocytes were lysed in Miki buffer (20 mM Tris, 1 mM EDTA, 4 mM $MgCl_2$, 10% glycerin, 1% Triton-X, and a cocktail of protease inhibitors, pH 8.0), homogenates were mixed with 45 µl of streptavidin-coupled Dynabeads, and bead-bound cell-surface proteins were purified through repeated washes. Detection of Lsi1 channels was carried out through chemiluminescence-based Western-blot analyses using a mouse anti-cMyc antibody (1/500) and a horseradish peroxidase-coupled secondary anti-mouse IgG (1/5000).
