# Supplementary material for: Si permeability of a deficient Lsi1 aquaporin in tobacco can be enhanced through a conserved residue substitution
Source: Plant Direct. 2019 Aug 21;3(8):e00163. doi: 10.1002/pld3.163 (PMC6702468; doi:10.1002/pld3.163)
Supplement: Supplementary file 4 [file PLD3-3-e00163-s006.pdf]

Table S1. Primers used throughout the study.

Experiment	Gene	Primers
qPCR	*NsLsi1* (*NsNIP2-1*)	Fwd: ATCGGACCTGTTTGTGGAAC<br>Rev: CGAGAATGACTGTCCTGGTG
	NsActin	Fwd: CCGTGGAGAAGAGCTACGAG<br>Rev: AGCTTCCATTCCGATCATTG
	NsEF1a1	Fwd: AGTATGCCTGGGTGCTTGAC<br>Rev: TCCAGGAGCATCAATCACAG
Gene cloning	*OsLsi1* (*OsNIP2-1*)	Fwd: ATGCATAGATCTATGGCCAGCAACAACTCGAGAAC<br>Rev: ATGCATACTAGTTCACACTTGGATGTTCTCCATCTC
	NsLsi1	Fwd: ATGCATAGATCTATGGAGAGTGAACGAGGAAACTC<br>Rev: ATGCATACTAGTTTATACGCCTTGTTCTTCATCATTG
	TaTIP2-1	Fwd: ATGCATAGATCTATGCCGGGCTCCATC<br>Rev: ATGCATACTAGTTTAGTAGTCGTTGCCGGCGA
Mutagenesis	*NsLsi1*^{P125F}	Fwd: TGGAGACAGGTACCATTYTATGCAGCAGCACAACTTAC<br>Rev: TGCTGCATARAATGGTACCTGTCTCCATGGGAAATGTC
Transient expression	*NsLsi1*^{WT} and *NsLsi1*^{P125F}	Fwd: CGCGGATCCATGGAGAGTGAACGAGGAAAC<br>Rev: GGACTAGTTTCTACGCCTTGTTCTTCATCATTG
