# Supplementary material for: Si permeability of a deficient Lsi1 aquaporin in tobacco can be enhanced through a conserved residue substitution
Source: Plant Direct. 2019 Aug 21;3(8):e00163. doi: 10.1002/pld3.163 (PMC6702468; doi:10.1002/pld3.163)
Supplement: Supplementary file 6 [file PLD3-3-e00163-s001.pdf]

Submission ID: 2019-00308

Title: Elevated Si transport and plasma-membrane localization is conferred by a conserved residue substitution in the Lsi1 aquaporin of tobacco

Authors: Devrim Coskun, Rupesh Deshmukh, Humira Sonah, S. M. Shivaraj, Rachelle Frenette-Cotton, Laurence Tremblay, Paul Isenring, and Richard Bélanger

REVIEWER COMMENTS FROM *PLANT JOURNAL* AND AUTHOR RESPONSES

Reviewer: 1

This manuscript was a pleasure to read; the topic is scientifically very relevant. The description of the results is very clear, but some points identified below could be further addressed in a revised version:

Figure 6 could be more informative regarding the localization of NsLsi1 in plasma membrane. A co-localization experiment with markers of the tonoplast would benefit the interpretation of the results (a TIP-RFP, for instance) and the quantification of fluorescence intensity could be performed.

We are unsure if/how testing for co-localization with a tonoplast-intrinsic protein would indeed "benefit the interpretation of the results", since, as is widely documented in the literature, Lsi1 aquaporins are strictly plasma-membrane intrinsic proteins. With that said, we can easily quantify fluorescence intensity in a revised version.

In any case, authors could discuss how a transient expression with a 35S promoter was the best approach to study differences between NsLsi1WT and NsLsi1P125F targeting to the plasma membrane.

Use of the ubiquitous 35S promoter is a very popular and straightforward method to test for the transient expression of genes, particularly those encoding membrane transporters. As such, this was the method we selected, and quite successfully, in our opinion, since we observed a clear membrane localization of the gene product, as well as differences between wildtype and mutant.

Furthermore, this result in N. benthamiana leaf cells is not a direct evidence (as acknowledged by the authors) that supports the observed increase of the transport activity, because transport experiments were performed in a different model (oocytes).

As the reviewer correctly notes, we did acknowledge that the leaf transient assay provided indirect evidence for the transport activity we observed (i.e. in oocytes).

In this regard, I do not totally agree with the sentence "Thus, this suggests the gain of function observed in oocytes is likely the result of increased protein abundance at the plasma membrane" (line 316), and results from western blot analysis in oocytes (even if the tagged protein is non-functional) could be more useful to support this conclusion.

We can revise the sentence in question to emphasize the indirect evidence the transient expression analysis provides. It would read as follows: "Thus, this suggests, albeit indirectly, that the gain of function observed in oocytes is possibly the result of increased protein abundance at the plasma membrane". Unfortunately, the results of the Western blot analysis would not be useful in this case, as not only were the tagged proteins non-functional, but yielded little/no membrane expression in blots, as explained in the text.

So, I am afraid the title of the paper could be rephrased to highlight only the observed change in the kinetic behavior of the encoded proteins, which, in my opinion, is a very interesting result. The altered transport kinetics mediated by NsLsi1P125F (involving a shift in both of Vmax and Km) relative to NsLsi1WT seems more related to a structural change than to an altered targeting of the protein.

Although we were careful not to imply that a single amino acid was responsible for the phenotypes observed in tobacco, we realize that the title may be misleading. Accordingly, we propose the following title change: Elevated Si transport and plasma-membrane localization is conferred by a conserved residue substitution in the Lsi1 aquaporin of tobacco

Figure 3 and 5 could be merged.

Given that both Figures 3 and 5 are based on the *Xenopus* oocyte assay, we understand why the reviewer may suggest we merge them. However, we believe that merger of these figures would create a disconnect with the narrative progression of the study. Figure 3 first demonstrates that the wildtype is non-functional. Only after the sequence alignment and *in-silico* analyses of Fig 4 and Table 1 do we isolate the amino-acid substitution of interest and thus perform the functional assay of Fig 5.

Reviewer: 2

1. If native NsLsi1 does not transport Si, what substrate it transports? Other substrates such as B, As, glycerol should also be tested.

This question, while interesting, is beyond the scope of our paper. Our objective was to assess Si permeability.

2. *Transformation of NsLsi1(P125F) into tobacco plants should be done to test whether it improves Si uptake.*

Transient leaf expression was performed for the purpose of localizing NsLsi1. To achieve higher uptake in tobacco, we surmise, would be dependent on myriad other factors (e.g. pre- and post-translational regulation, presence of functional downstream Si transporters like Lsi2, etc.). Incidentally, a similar finding reported in *Plant Journal* (https://onlinelibrary.wiley.com/doi/pdf/10.1111/j.1365-313X.2011.04483.x) was not subjected to the process of transformation for the purpose of Si uptake in plants.

3. The transport activity of NsLsi1(P125F) for Si was lower than rice Lsi1. What is the reason for this?

Quantitative comparisons are always speculative in absence of a strong method to compare protein quantities in oocytes. The simple fact that our results show a gain of function is already a difficult feat to achieve.

4. Other point-mutation shown in Table 1 should also be tested, not just based on speculation.

Testing of P125F was not based on speculation but on *in-silico* predictions as shown in Table 1. As it turned out, the prediction was accurate although we do not eliminate the possibility that other residues may also affect permeability to a lesser extent.

5. Fig. 5, it is inappropriate to calculate Km and Vmax because NsLsi1 is a channel-type transporter. Different from other type transporters, it does not fit Michaelis-Menten regression.

Channel-type transporters can follow Michaelis-Menten kinetics, particularly when patch-clamp is avoided, and thus regression can sometimes apply (e.g.https://aip.scitation.org/doi/abs/10.1063/1.1522709; https://www.pnas.org/content/103/31/11446.short**). Nevertheless, we are aware that Michaelis-Menten kinetics strictly apply to enzyme-substrate interactions so the term Michaelis-Menten-*like* kinetics could be used. In addition, Reviewer 1 was perfectly fine with our approach and considered it a strong point of this paper.**

6. Fig. 1C and Fig. 4 are displayed in duplicate. Should delete one of them.

Fig 1C and 4 serve different purposes. The former is a direct comparison with 1A and 1B, demonstrating the conundrum with tobacco, whereas the latter highlights the putative residues that contrast with those found in 12 other species.
