# Supplementary material for: Si permeability of a deficient Lsi1 aquaporin in tobacco can be enhanced through a conserved residue substitution
Source: Plant Direct. 2019 Aug 21;3(8):e00163. doi: 10.1002/pld3.163 (PMC6702468; doi:10.1002/pld3.163)
Supplement: Supplementary file 5 [file PLD3-3-e00163-s002.docx]

Dear Dr. Mendoza-Cozatl,

We thank you and the reviewers for your constructive handling of our manuscript. We were pleased to learn that both reviewers largely viewed our work positively. They provided many valuable suggestions, which we address in detail below, and have made several key changes accordingly. We believe the work is significantly improved as a result.

With regards to the two main points you raised:

(1) Quantitative analysis of the confocal micrographs has now been performed, along with appropriate statistics (see the revised Fig. 6, Suppl. Fig. 1). As you will see, the results were unequivocal: the P125F substitution corresponded with a nearly 3-fold increase (relative to WT; p < 0.0001) in GFP fluorescence localized at the plasma membrane (PM).
(2) While we agree that an independent assay to confirm our conclusions regarding NsLsi1 PM expression would be ideal, as we discussed in our original submission, numerous Western-blot attempts (with three independent epitopes tested at either terminal) in the *Xenopus* oocyte system were unsuccessful, yielding non-functional channels and little/no indication of membrane expression. It is not uncommon in the oocyte system for a foreign protein to become less functional or even non-functional when an epitope is added to either terminal (likely due to protein-processing or conformational issues). We have made this observation on numerous occasions in the past with mammalian proteins. Given the futile attempts at changing the nature of the epitope, it is quite unlikely, based on R2’s suggestion, that the outcome will change with the use of a (larger) GFP tag. For this reason, and given the amount of additional work that would be required to generate a NsLsi1-GFP (WT and mutant) construct in the appropriate oocyte expression vector, we were hoping that the localization data presented *in planta* would be sufficient, all the more so that the data in question are now extremely convincing based on quantitative analysis. However, to mitigate the criticism, we acknowledge that the original title was perhaps too strong and have thus revised it and eliminated the inference to plasma-membrane localization. The title is now: *Si permeability of a deficient Lsi1 aquaporin in tobacco can be enhanced through a conserved-residue substitution*.

We hope you agree with these changes and look forward to your assessment.

Sincerely,

The Authors

Responses to Reviewer #1:

- Re Si measurements: As per our Introduction, it is well established that a functional, Si-permeable, Lsi1 (in coordination with Lsi2) is necessary for xylem loading of Si, and thus Si accumulation in shoot tissues (Ma & Yamaji, 2015, *Trends Plant Sci* **20**: 435). It is in the shoots that clear phenotypic differences with respect to Si content manifest across species (Hodson et al., 2005, *Ann Bot* **96**: 1027). For these reasons, we provide shoot rather than root data. To be clear, the tissue data was not used in support of any arguments surrounding the permeability of NsLsi1 from tobacco; such arguments were based on functional assays in the *Xenopus* oocyte system.
- Re qPCR: The question of *Lsi1* gene expression responses to plant Si status appear to be species dependent and highly variable (see e.g. Ma et al., 2006, *Nature* **440**: 688; Montpetit et al., 2012, *Plant Mol Biol* **79**: 35; Vulavala et al., 2016, *Planta* **243**: 217). In general, no clear and conclusive trend has ever been linked with *Lsi1* expression and Si and our results reflect this phenomenon
- Re Fig. 4: Fig. 4 was not consistent with Fig. 1 in terms of species comparison simply because the two figures addressed different research questions. Fig. 1 demonstrates that those species with a GSGR selectivity filter and 108 amino acids spanning the NPA domains in their Lsi1 corresponded with high tissue Si content (except for tobacco – the basis of our study). Fig. 4 attempts to identify residues of interest related to tobacco’s phenotype by comparing with numerous *functional* homologs from Si-accumulating species; thus, SlLsi1 from tomato was omitted, as it is a Si-impermeable Lsi1 from a non-accumulating species. The reviewer made a very astute observation and correctly notes, however, that SlLsi1 contains a valine (V) at the residue position of interest. This point is now addressed in our Discussion (see Ln 344-351).
- Re Figs. 5 and 6: We thank the reviewer for reading the MS carefully and appreciating the difficulty in working with oocytes. The suggestion for looking at the effect of other substitutions in tobacco (the P125V in particular) is valid and we are currently planning additional experiments along these lines. However, these additional experiments will require several months, if not close to a year, such that it will not be possible to provide new data in a reasonable period of time. In addition, we do not think that the outcome of these additional studies will invalidate the conclusion of the current study. For these reasons, we hope you would agree that we limit ourselves currently to the variants already included in the submission.
- As mentioned above, quantification analysis was performed on confocal micrographs, and the details regarding the analysis and statistics were provided in the revised MS (see Ln 202-208, 281-282, 325-327).

Responses to Reviewer #2:

- We were careful not to imply that the residue of interest, alone, causes the low Si permeability of NsLsi1 and the low Si content *in planta*. Instead, by demonstrating that the P125F substitution resulted in a gain of function, which we hypothesize to be attributed to increased plasma-membrane localization, we aimed to simply further characterize the molecular determinants of Si permeability in plants, which is by no means completely understood.
- As above, the quantification of GFP fluorescence has now been included. We thank the reviewer for raising this critical point.
- WT NsLsi1 is clearly expressed at the cell surface as shown in Figs. 3 and 5 (where the Si content from water-injected oocytes have been subtracted out), and as shown in our original Fig. S1, but has much lower Si permeability than OsLsi1. In addition, the oocyte system did provide us with insight regarding Si permeability and channel function: despite the differences in V_max_, there were also differences in K_m_ as demonstrated by our kinetic analyses of Figs. 3 and 5.
- Re Fig. 5: The Discussion points related to possible structural changes were speculative, based on changes observed in substrate affinity between mutant and wildtype proteins. As demonstrated in our original Fig. S1, even after 3 h of incubation in Si uptake solution, TaTIP2-1-expressing oocytes failed to accumulate Si above background levels. This is particularly striking when comparing TaTIP2-1 to NsLsi1, and especially to OsLsi1. Furthermore, considering that TaTIP2-1 does not have the molecular signatures of a Si-permeable channel as defined in our Introduction, and considering that oocytes are rarely unable to process exogenous WT proteins adequately, we think it is safe to conclude that TaTIP2-1 is not Si-permeable. This result corroborates the wealth of literature supporting the specificity of NIP-III aquaporins with respect to Si permeability.
- Although the Reviewer raises a valid suggestion regarding GFP-antibodies, as discussed above, we have little confidence in this pursuit, based on several similar yet futile attempts. Moreover, although the Reviewer raises interesting questions regarding the mechanism(s) underlying the PM localization (or lack thereof), these questions are beyond the scope of the present study but will surely merit future investigations.
